# Protein Tyrosine Phosphatase Receptor Type D Regulates Neuropathic Pain After Nerve Injury *via* the STING-IFN-I Pathway

**DOI:** 10.3389/fnmol.2022.859166

**Published:** 2022-04-14

**Authors:** Chengkuan Sun, Guangzhi Wu, Zhan Zhang, Rangjuan Cao, Shusen Cui

**Affiliations:** Department of Hand Surgery, China-Japan Union Hospital of Jilin University, Changchun, China

**Keywords:** protein tyrosine phosphatase receptor type D, neuropathic pain, chronic constriction injury, stimulator of interferon genes, type I interferon

## Abstract

Neuropathic pain is usually caused by injury or dysfunction of the somatosensory system, and medicine is a common way of treatment. Currently, there are still no satisfactory drugs, like opioids and lidocaine, which carry a high risk of addiction. Protein tyrosine phosphatase receptor type D (PTPRD) is a known therapeutic target in addiction pathways and small molecule inhibitors targeting it, such as 7-butoxy illudalic acid analog (7-BIA), have recently been developed to tackle addition. PTPRD is also upregulated in the dorsal root ganglion (DRG) in a rat model of neuropathic pain, but is not yet clear whether PTPRD contributes to the development of neuropathic pain. Here, we established a chronic constriction injury (CCI) and evaluated PTPRD expression and its association with neuropathic pain. PTPRD expression was found to gradually increase after CCI in DRGs, and its expression was concomitant with the progressive development of hypersensitivity as assessed by both mechanical and thermal stimuli. Both PTPRD knockdown and administration of PTPRD inhibitor 7-BIA alleviated CCI-induced neuropathic pain while upregulating STING and IFN-α in the DRG. Treatment with H-151, a STING inhibitor, abolished the analgesic effects of PTPRD knockdown. Taken together, our study suggests that increased levels of PTPRD in the DRG following CCI are involved in the development of neuropathic pain *via* the STING-IFN-I pathway. 7-BIA, a small molecule inhibitor of PTPRD with anti-addiction effects, may represent a novel and safe therapeutic strategy for the clinical management of neuropathic pain without the risk of addiction.

## Introduction

Neuropathic pain is a type of chronic pain caused by injury or dysfunction of the somatosensory system and can dramatically influence the quality of the life of affected patients (Baron et al., 2010; Cohen and Mao, 2014; Braden et al., 2020). Neuropathic pain is estimated to constitute a fifth of all chronic pain cases (Fillingim et al., 2016; Bouhassira, 2019). Diabetes, nerve compression, infection, or trauma, neuroma, and autoimmune diseases causing ectopic neural activity in the dorsal root ganglion (DRG) or dorsal horn of the spinal cord may induce neuropathic pain (Cohen and Mao, 2014; Wang et al., 2018; Dai et al., 2020). A number of previous studies investigating the mechanisms underlying neuropathic pain (Campbell and Meyer, 2006; Bannister et al., 2020) indicated that the DRG plays a vital role in the progression of neuropathic pain in the peripheral nerve system (Berta et al., 2017). Following peripheral nerve injury, a series of pathological changes involved in sensitization of nociceptive pathways occur in the DRG, including activation of ion channels, glial, and immune cells (Scholz and Woolf, 2007; Yeh et al., 2020; Finnerup et al., 2021). Treatment of neuropathic pain remains a challenge, as patients often only experience insufficient pain relief. This might be due to a heterogeneity of chronic neuropathic pain mechanisms. Additionally, some patients prescribed opioids such as morphine exhibit severe side effects, including drug addiction (Cooper et al., 2017). Therefore, novel satisfactory treatment options for the clinical management of neuropathic pain are urgently required.

Protein tyrosine phosphatase receptor type D (PTPRD), a member of the leukocyte common antigen-related receptor (LAR) family located on human chromosome 9, was first discovered in 1990 (Krueger et al., 1990). PTPRD is a transmembrane protein with extracellular immunoglobulin and fibronectin domains and contributes to cell adhesion and synaptic specificity (Pulido et al., 1995; Song et al., 2016). Previous studies have shown that PTPRD may be involved in several disorders of the central nervous system, including Alzheimer’s disease, mood lability, restless legs syndrome, and vulnerability to addiction (Wang and Bixby, 1999; Ensslen-Craig and Brady-Kalnay, 2004; Vellieux and d’Ortho, 2020). Inhibition of the phosphatase activity of PTPRD has previously been shown to reduce addiction to cocaine (Uhl et al., 2018), suggesting that PTPRD could represent a therapeutic target for addiction disorders.

Interestingly, PTPRD is also significantly upregulated in DRGs following chronic constriction injury (CCI) in rats (Cao et al., 2019; Sun et al., 2020). CCI represents a classical model for neuropathic pain, following which animals exhibit hypersensitivity to mechanical and thermal stimuli (Decosterd and Woolf, 2000; Challa, 2015; Gopalsamy et al., 2019; Guida et al., 2020). Despite initial insights into an upregulation of PTPRD in DRGs following CCI, the temporal dynamics of expression changes and whether PTPRD was involved in development and onset neuropathic pain remained unknown. In this study, we found that PTPRD expression gradually increased in DRG after CCI in mice, consistent with previous reports in rats, and PTPRD upregulation coincided with the onset of hypersensitivity. PTPRD inhibition using shRNA or small molecule inhibitor 7-butoxy illudalic acid analog (7-BIA) ameliorated neuropathic pain which suggested that PTPRD played a key role in mediating neuropathic pain following nerve injury. As PTPRD has previously been identified as a target for addiction treatment, our study suggests that it may represent a safe analgesic therapeutic strategy for the clinical management of neuropathic pain with low addiction risk.

## Materials and Methods

### Animals

All animal experiments were approved by the Institutional Animal Care and Use Committee in Jilin University and complied with relevant ethical guidelines. C57BL/6 male mice weighing 18–20 g were purchased from the Animal Centre of Jilin University. All mice were housed at 25°C in a humidity-controlled room on a 12 h light–dark cycle with *ad libitum* to food and water. Experiments were conducted on mice aged between 8 and 12 weeks old. Animals were randomly assigned to a treatment group and were allowed to acclimatize to experimental conditions for 3 days before the experiments.

### Reagents and Drug Delivery

Our study used H-151 (10 nM, MedChemExpress, HY-112693) and 7-BIA (10 or 20 mg/kg, MedChemExpress, HY-115496). Both reagents (H-151, 7-BIA) were diluted in 10% DMSO and 90% corn oil. H-151 was administered *via* intrathecal injection. All intrathecal injections were performed under brief anesthesia induced by isoflurane. Briefly, a small area was shaved on the back of the animal and a spinal puncture was carried out at L4–L5 using a microsyringe needle to deliver a maximum of 10 μl reagents into the subarachnoid space. A tail movement confirmed a successful intrathecal injection. 7-BIA was administrated by intraperitoneal injection.

### Chronic Constriction Injury

The CCI neuropathic pain model was induced in 8- to 10-week-old male C57 mice under isoflurane anesthesia, as previously described (Bennett and Xie, 1988; Gopalsamy et al., 2019). Briefly, the right thigh was shaved and sterilized with iodophor. A transverse incision was made under the long head of the biceps femoris and the sciatic nerve was exposed *via* blunt dissection. Under a microscope, three ligatures (8-0 Prolene) were placed around the sciatic nerve 1 mm apart from proximal to the trifurcation. Ligatures were loosely tied to prevent an arrest of the epineural blood flow. In the sham group, the same procedure was performed without ligation of the sciatic nerve.

### Behavioral Testing in Mice

All sensory behavioral testing in mice was performed between 9 am and 6 pm in an isolated experimental room maintained at 21–25°C. Mice were placed in the experimental room at least 3 day prior to baseline behavioral testing and all tests were performed by the same experimenter who was blinded to treatment. To assay mechanical sensitivity, the paw withdrawal threshold (PWT) of the hind paw was recorded using a series of von Frey filaments (from 0.16 to 2.0 g, Aesthesio, UGO) by perpendicular stimulation of the central plantar surface of the paw at resting state. A positive response was recorded if the paw was sharply withdrawn. To test the 50% PWT, we used the up–down method, as previously described (Michaelidou et al., 2013). Typically, 0.16 g filaments do not elicit paw withdrawal in mice and were used to test the paw withdrawal frequency (PWF). Mice were repeatedly stimulated 10 times, in 1 min intervals (Aesthesio, UGO).

To assay temperature sensitivity, thermal paw withdrawal latency (PWL) was assessed using a hot plate (BIO-CHP-ER, Bioseb). Briefly, the mice were placed on a 52°C metallic plate surrounded by an acrylic container. A sensory response was recorded by flinching, licking one of the hind paws, or jumping. The test was stopped if the paw was not withdrawn within 20 s. The hot plate test was repeated three times for each mouse, with at least 20 min intervals between each repetition.

Motor coordination was evaluated by rotarod testing (Panlab, LE8505), as previously described (Bohlen et al., 2009). Rotarods were accelerated from 4 to 40 rpm over 300 s. Over the course of 3 days, each mouse underwent three trials per day separated by 15 min intervals. The fall latency was recorded on the third day.

### Lentiviral Transduction

Two different target shRNAs were designed using BLOCK-iT™ RNAi Designer and cloned into the lentiviral EGFP-expressing PLL3.7 plasmid. The shRNA sequences were as follows: shPTPRD1, 5′-GGTTCAGATGACTCCGGTTAC-3′; shPTPRD2, 5′-GGTTGAAAGCAAATGATAA-3′. For lentiviral packaging, plasmids containing shRNAs (including shRNAs against PTPRD and scrambled shRNA) were transfected into the packaging cell line 293T with the packaging plasmid psPAX2 (#12260, Addgene) and the envelop plasmid pMD2.G (#12259, Addgene) at a ratio of 4:3:1. Viruses were harvested 48 or 72 h later by filtering cell lysates with 0.45 μm filters (Millipore) followed by ultracentrifugation for 4 h at 25,000 rpm (Beckman SW28 rotor) and resuspension in phosphate-buffered saline (PBS, 50 μl). The efficiency of the two shRNAs in 293T cells was tested using quantitative real-time PCR (qRT-PCR) and Western blotting.

### Dorsal Root Ganglion Injection

DRG injections were performed as previously described (Fischer et al., 2011). L4 and L5 vertebrae were exposed by a 2 cm longitudinal incision and blunt dissection of multifidus and longissimus lumborum muscles. The processus accessorius and parts of the left processus transversus were removed to expose L4/L5 DRGs. The spinal column was fixed in a stereotaxic frame and lentiviruses (300 nL for each DRG) were injected at 30 nL/min using a 36 G NanoFil needle on a NanoFil syringe, controlled by a micropump (World Precision Instruments, Sarasota, FL, United States).

### Immunohistochemistry

Mice were deeply anesthetized with isoflurane and perfused *via* injection of 20 mL PBS followed by 4% paraformaldehyde (PFA) into the ascending aorta. After perfusion, L–L5 DRGs were removed and fixed in the pre-cooled 4% PFA. DRG samples were dehydrated in a 30% sucrose solution, and 12 μm transverse sections were cut using a cryostat microtome (CM1950, Leica, Germany). After washing sections three times in PBS, tissues were permeabilized in 1% Triton X-100 at room temperature and blocked with 5% BSA in PBS containing 0.5% Tween 20 (PBS-T). Samples were incubated with the following primary antibodies overnight at 4°C: rabbit anti-PTPRD (1:200; Novus, NBP2-94767), rabbit anti-NeuN (1:100, CST, D4G40), mouse anti-Tuj1 (1:1000, Abcam, 2G10), mouse anti-S100β (1:1000, Sigma, S2532), or mouse anti-GFAP (1:200, Abmart, MB0345S). The following day, sections were washed three times in PBS-T, and primary antibodies were visualized using secondary antibodies labeled with Alexa-488 and Alexa-546 (1:800; Invitrogen). Fluorescence (DM4B, Leica, Germany) and laser scanning confocal microscopes (A1HD25, Nikon, Japan) were used to capture images.

### Western Blotting

Mice were deeply anesthetized with isoflurane and L4–L5 DRGs were dissected and placed in RIPA assay lysis buffer (C50008, Sangon Biotech). Protein concentration was measured using a bicinchoninic acid (BCA) assay (P0010, Beyotime Biotechnology, China). Proteins were separated by 10% SDS-PAGE and transferred onto PVDF membranes. PVDF membranes were blocked for 90 min at room temperature using 5% BSA in TBS-T and subsequently probed with the following primary antibodies at 4°C overnight: rabbit anti-PTPRD (1:1000, Novus, NBP2-94767), rabbit anti-STING (1:1000, CST, D2P2F), or mouse anti-GAPDH (1:1000, TransGen Biotech). The membranes were subsequently incubated with horseradish peroxidase (HRP)-conjugated secondary antibodies (1:1000, Beyotime) for 120 min at room temperature. Finally, images were acquired using a GS800 Densitometer Scanner. The optical density of protein bands was quantified using ImageJ, with GAPDH used as the loading control to normalize protein expression levels. Each experiment was repeated three times.

### Quantitative Real-Time PCR Analysis

Total RNA from L4 to L5 DRGs or cells was extracted using the Eastep™ Super Total RNA Extraction Kit (LS1040, Promega), and cDNA was synthesized using the Tranc-Script One-Step cDNA Synthesis SuperMix (AT311, TransGen Biotech, China). Primers (shown in Supplementary Table 1) were synthesized by Genewiz Biotech. For PCR reactions, the TB Green™ Premix Ex Taq™ (RR420A, TaKaRa) mix was used. PCR reactions were carried out on a Real-Time PCR System (CFX96, Bio-Rad, United States), and the 2^–Δ^ ^Δ^ *^Ct^* method was used to calculate relative mRNA expression. mRNA levels were normalized to *Gapdh*.

### Enzyme-Linked Immunosorbent Assay

Levels of inflammatory cytokines IL-6, IL-1β, TNF-α, IL-10, and IFN-α in DRG tissues were determined by enzyme-linked immunosorbent assay (ELISA) according to the manufacturer’s instructions (Anoric Bio-technology Co., Ltd., Tianjin, China). Absorbance at 450 nm was measured using a microplate reader (Thermo Fisher K3, United States). Each experiment was performed three times.

### Statistical Analysis

Statistical analysis was performed using SPSS 21.0 for windows (SPSS, Inc., Chicago, IL, United States). All data are presented as mean ± SEM. Behavioral data were analyzed using two-way ANOVAs followed by *post hoc* Tukey’s tests to compare multiple groups. Unpaired Student’s *t*-tests were used for comparisons between two groups. All experiments were performed at least three times independently. *P*-values less than 0.05 were considered statistically significant. Asterisks correspond to the following significance levels: *p < 0.05, ^**^p < 0.01, ^***^p < 0.001.

## Results

### Protein Tyrosine Phosphatase Receptor Type D Expression Is Increased in DRGs After Chronic Constriction Injury

**FIGURE 1:**
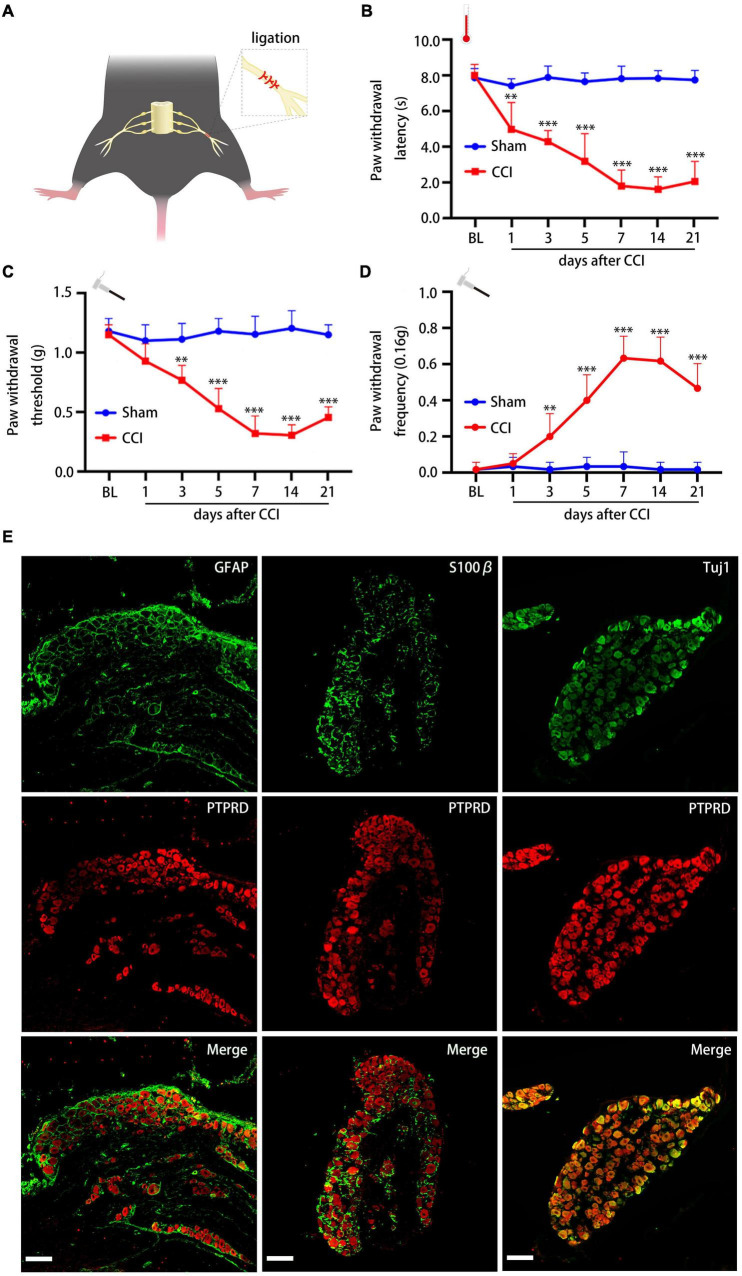
Chronic constriction injury induces neuropathic pain and PTPRD is expressed in DRG neurons. **(A)** Schematic overview of the CCI-induced neuropathic pain model. **(B–D)** Paw withdrawal latency (PWL), paw withdrawal threshold (PWT), and paw withdrawal frequency (PWF) before the surgery (BL) and 1, 3, 5, 7, 14, and 21 days after CCI. *N* = 6 mice per group, ^**^p < 0.01, ^***^p < 0.001. **(E)** Representative images of PTPRD co-staining with GFAP, S100β, or Tuj1 in DRG sections 7 days after CCI. Scale bars, 100 μm. Statistical comparisons were performed by two-way ANOVA with Tukey’s *post hoc* test **(B–D)**.

RNA sequencing (RNA-seq) experiments previously indicated that PTPRD is upregulated in rat DRGs 14 days after CCI (Zhou et al., 2017; Cao et al., 2019; Sun et al., 2020). To confirm these findings in mice and further investigate the role of PTPRD in neuropathic pain, we established a mouse CCI model (Figure 1A). Behavioral testing confirmed that the hot plate test PWL was decreased after CCI surgery, indicating that the induction of the neuropathic pain model was successful. Likewise, von Frey filament tests demonstrated that the PWT decreased, while the PWF increased, from day 3 to day 21 after CCI surgery (Figures 1B–D). The above behavioral results indicated a successful induction of neuropathic pain in mice following CCI.

We next evaluated which cells in the DRG expressed PTPRD by co-staining sections from 7 days post CCI with PTPRD and astrocyte marker GFAP, satellite glial cell marker S100β, or neuronal marker Tuj1. This revealed that PTPRD was exclusively expressed in DRG neurons (Figure 1E).

**FIGURE 2:**
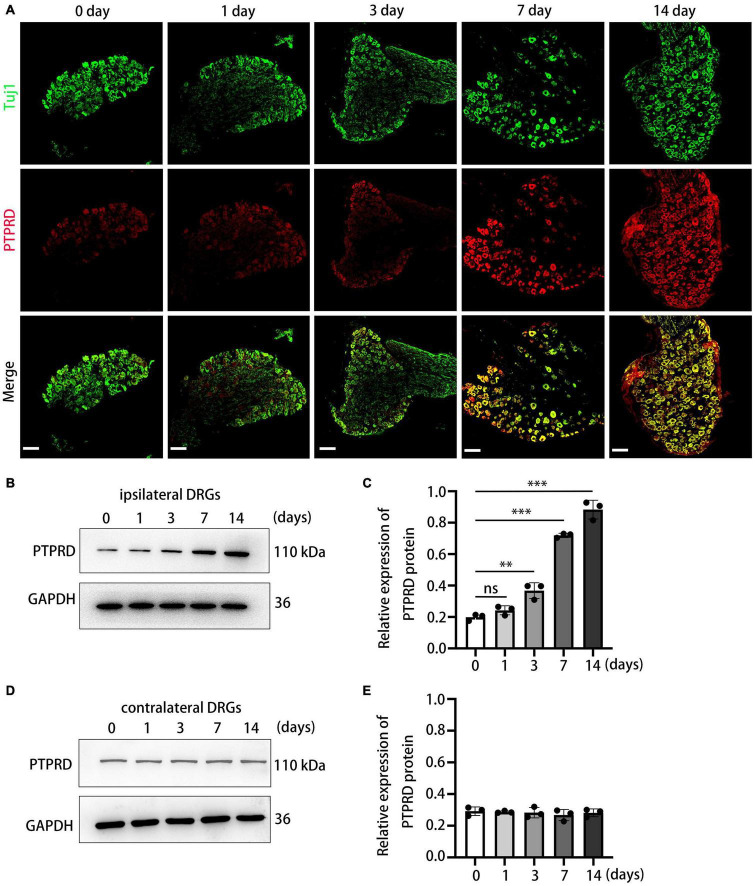
Protein tyrosine phosphatase receptor type D expression levels are increased in the DRG after CCI. **(A)** Representative images of DRG sections stained for PTPRD (red) and Tuj1 (green) in the indicated groups. Scale bars, 100 μm. **(B)** Western blot of PTPRD levels in ipsilateral DRGs (L4–L5) at 0, 1, 3, 7, and 14 days after CCI. Each experiment was repeated three times. **(C)** Quantitative analysis of data in **(B)**. *N* = 3, ^**^p < 0.01, ^***^p < 0.001. **(D)** Western blot of PTPRD levels in contralateral DRGs (L4–L5) at 0, 1, 3, 7, and 14 days after CCI. Each experiment was repeated three times. **(E)** Quantitative analysis of data in **(D)**. *N* = 3, *p* > 0.05. Statistical comparisons were performed by unpaired Student’s *t*-test **(C,E)**.

To investigate the temporal dynamics of PTPRD expression in more detail, we conducted immunohistochemical staining on sections collected before surgery (day 0) and on days 1, 3, 7, and 14 after CCI surgery. PTPRD exhibited only low expression in the uninjured DRG (before surgery), but expression significantly increased by day 7 and 14 after CCI (Figure 2A). We next validated our immunohistochemistry results by Western blotting. Western blotting indicated that PTPRD expression was slightly elevated by day 3 and significantly increased by days 7 and 14 after CCI (Figures 2B,C). Importantly, the expression of PTPRD in contralateral DRGs did not change and was maintained at basal levels for each timepoint we evaluated (Figures 2D,E). These results suggested a potential correlation between elevated PTPRD protein levels and the onset of neuropathic pain.

### Knockdown of Protein Tyrosine Phosphatase Receptor Type D Ameliorates Chronic Constriction Injury-Induced Neuropathic Pain

**FIGURE 3:**
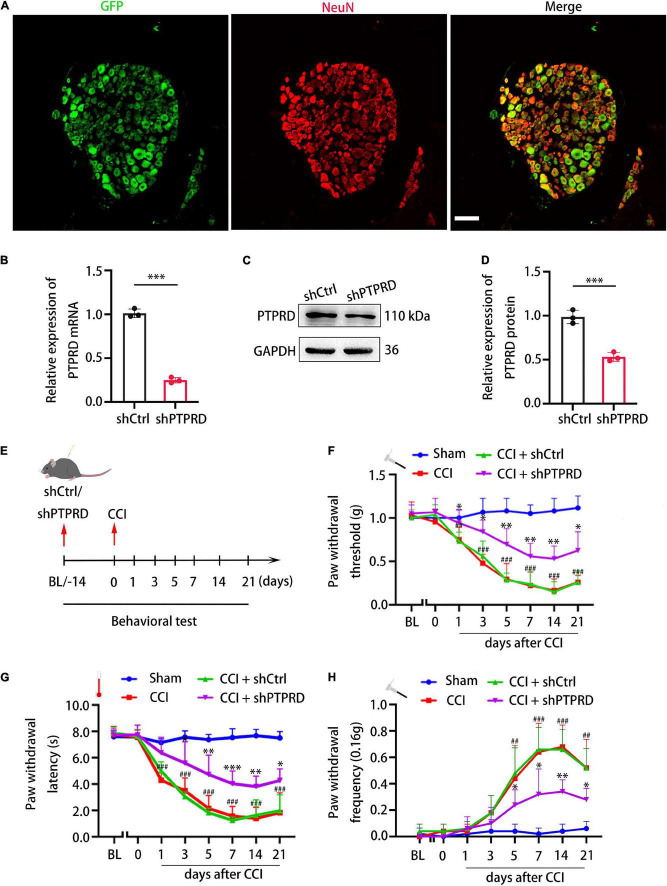
Protein tyrosine phosphatase receptor type D knockdown attenuates neuropathic pain following CCI. **(A)** Representative images of GFP (green) and NeuN staining (red) 14 days after shPTPRD injection in the DRG. Scale bar, 100 μm. **(B)** qRT-PCR analysis of the relative expression of PTPRD in DRGs transfected with shCtrl or shPTPRD after CCI. *N* = 3, ^***^p < 0.001. **(C)** Western blot of PTPRD in DRG tissues transfected with shCtrl or shPTPRD after CCI. Each experiment was repeated three times. **(D)** Quantitative analysis of data in **(C)**. GAPDH served as the loading control. *N* = 3, ^***^p < 0.001. **(E)** Schedule of lentivirus administration, surgery, and behavioral testing. **(F–H)** Paw withdrawal threshold, paw withdrawal latency, and paw withdrawal frequency prior to virus injection (BL), on the day of surgery, or 1, 3, 5, 7, 14, and 21 days after CCI. *N* = 6 mice per group. CCI + shPTPRD vs. CCI + shCtrl, *p < 0.05, ^**^p < 0.01, ^***^p < 0.001. CCI vs. sham, ^#^*p* < 0.05, ^##^*p* < 0.01, ^###^*p* < 0.001. Statistical comparisons were performed using unpaired Student’s *t*-test **(B,D)** or two-way ANOVA with Tukey’s *post hoc* test **(F–H)**.

To confirm whether PTPRD participated in the development neuropathic pain following CCI, we generated lentiviruses containing one of two PTPRD-targeting shRNAs (shPTPRD-1 and shPTPRD-2) or a control shRNA (shCtrl). The silencing efficiency of the two shPTPRDs was assessed by qRT-PCR (Supplementary Figure 1A) and Western blotting (Supplementary Figures 1B,C), and shPTPRD-2 was chosen for subsequent experiments as it performed better. Viruses containing shCtrl or shPTPRD-2 (shPTPRD in short hereafter) were injected into L4-L5 DRGs. DRGs were isolated and stained 14 days after transfection, which revealed that the majority of NeuN^+^ DRG neurons were successfully transfected (Figure 3A). qRT-PCR confirmed an *in vivo* PTPRD mRNA silencing efficiency of 75.11 ± 2.55% (Figure 3B). The silencing ability of shPTPRD was further tested by Western blotting, which showed a 46.83 ± 4.12% decrease in PTPRD protein levels (Figures 3C,D). These results showed that the lentiviral shRNA delivery successfully knocked down RNA and protein levels of PTPRD in DRG tissues *in vivo*.

We next went on to assess the impact of PTPRD knockdown on development of neuropathic pain. Fourteen days before CCI, baseline performance (BL) in the von Frey and heat plate tests was recorded, after which lentiviruses containing shRNAs were administered. After injection, mice were allowed to recover for 14 days. Behavioral tests were then again conducted prior to CCI surgery (day 0) or 1, 3, 5, 7, 14, and 21 days after surgery (Figure 3E). shPTPRD did not influence mechanical or thermal sensitivity prior to CCI, with animals showing similar PWT, PWL, and PWF compared to shCtrl-injected animals 14 days after lentiviral injection, which suggested that PTPRD did not participate in modulating or inducing neuropathic pain under physiological condition. In the shCtrl group, mechanical and thermal hypersensitivity were observed as early as 1 day after CCI and lasted for 21 days, when animals were sacrificed. Conversely, PTPRD silencing significantly attenuated mechanical and thermal hypersensitivity (Figures 3F–H). Knockdown of PTPRD did not alter motor behaviors (Supplementary Figure 2A). The above results suggested that upregulation of PTPRD participated in the development of neuropathic pain following CCI in mice.

### Protein Tyrosine Phosphatase Receptor Type D Knockdown Increases IFN-α Levels in the Dorsal Root Ganglion After Chronic Constriction Injury

**FIGURE 4:**
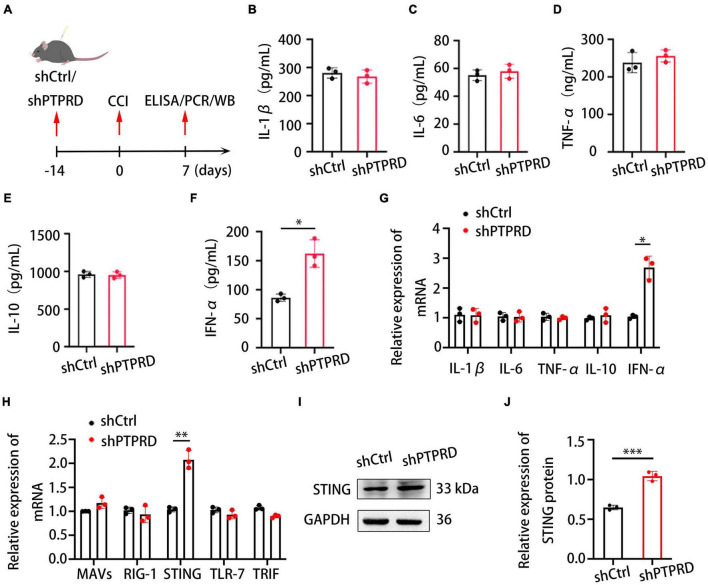
Protein tyrosine phosphatase receptor type D knockdown increases IFN-α and STING levels in the DRG after CCI. **(A)** Schedule of lentivirus administration, surgery, ELISA, qRT-PCR, and Western blotting. **(B–F)** ELISA assessing IL-1β, IL-6, TNF-α, IL-10, and IFN-α levels in the DRG after shCtrl or shPTPRD virus injection. CCI + shPTPRD vs. CCI + shCtrl, *N* = 3. For IFN-α, *p < 0.05. **(G)** qRT-PCR analysis of the relative expression of IL-1β, IL-6, TNF-α, IL-10, and IFN-α in the DRG after shCtrl or shPTPRD virus injection. CCI + shPTPRD vs. CCI + shCtrl, *N* = 3. For IFN-α, *p < 0.05. **(H)** qRT-PCR analysis of the relative expression of MAVS, RIG-1, STING, TLR-7, and TRIF in the DRG after shCtrl or shPTPRD administration. CCI + shPTPRD vs. CCI + shCtrl, *N* = 3. For STING, ^**^p < 0.01. **(I)** Western blot evaluation of the DRG levels of STING after shCtrl or shPTPRD treatment. Experiment was repeated three times. **(J)** Quantitative analysis of data in **(H)**. CCI + shPTPRD vs. CCI + shCtrl, *N* = 3, ^***^p < 0.001. Statistical comparisons were performed by unpaired Student’s *t*-test **(B–H,J)**.

We subsequently aimed to investigate the molecular mechanism through which PTPRD contributed to the development of neuropathic pain. Previous studies suggested that inflammation and the balance of pro- and anti-inflammatory cytokines play an important role in regulating neuropathic pain in injured nerves (Xu et al., 2018; Zheng et al., 2019). We hypothesized that the amelioration of neuropathic pain following PTPRD knockdown in CCI mice was associated with changes in neuroinflammation. To test this hypothesis, we evaluated levels of multiple inflammatory cytokines 7 days after CCI using ELISA (Figure 4A). DRG levels of IL-6, IL-1β, TNF-α, and IL-10 were not significantly altered following PTPRD knockdown (Figures 4B–E). Conversely, levels of IFN-α were significantly increased (Figure 4F) compared to shCtrl DRGs. These results were confirmed by qRT-PCR (Figure 4G). Based on this finding, we aimed to determine how PTPRD was involved in the regulation of IFN-α expression in the DRG after CCI. Previous reports have described that mitochondrial antiviral signaling protein (MAVs), acid-inducible gene I (RIG-I), stimulator of interferon genes (STING), Toll-like receptor-7 (TLR-7), and Toll/IL-1 receptor domain-containing adaptor (TRIF) can promote the release of IFN-1. We therefore assessed mRNA levels of these potential upstream regulators by qRT-PCR and found that the expression of STING, but not MAVs, RIG-1, TLR-7, or TRIF, was increased after PTPRD knockdown (Figure 4H). Consistently, Western blotting also demonstrated an enhanced protein expression of STING in shPTPRD DRGs (Figures 4I,J).

### Protein Tyrosine Phosphatase Receptor Type D Regulates Chronic Constriction Injury-Induced Neuropathic Pain *via* the STING-IFN-I Pathway

**FIGURE 5:**
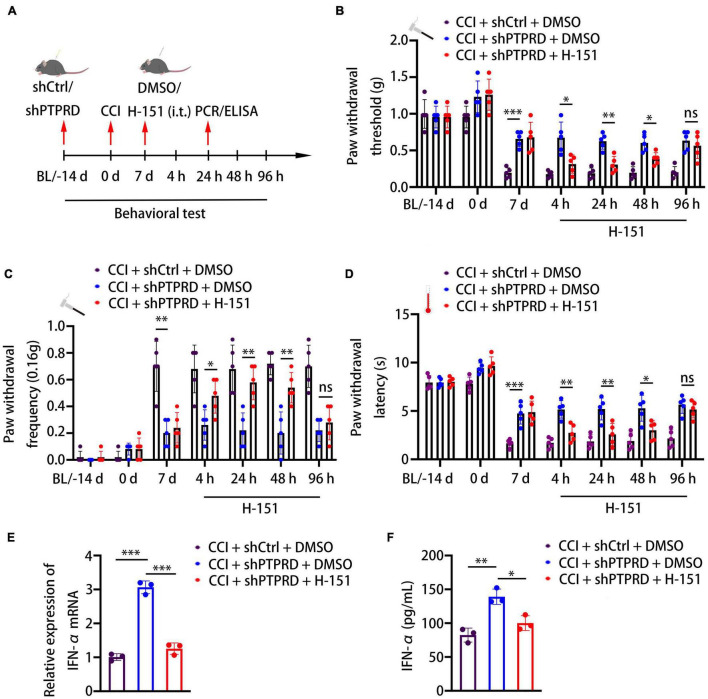
Protein tyrosine phosphatase receptor type D regulates CCI-induced neuropathic pain *via* the STING-IFN-α pathway. **(A)** Schedule of the virus injection, surgery, STING inhibitor (H-151, 10 nM, i.t.) administration, behavioral testing, qRT-PCR, and ELISA. **(B–D)** Paw withdrawal threshold, paw withdrawal latency, and paw withdrawal frequency before virus injection (BL), the day of surgery, 7 days after the surgery, or 4, 24, 48, and 96 h after H-151 treatment. *N* = 5 mice per group, *p < 0.05, ^**^p < 0.01, ^***^p < 0.001. **(E)** qRT-PCR analysis of the relative expression of IFN-α. *N* = 3, ^***^p < 0.001. **(F)** ELISA analysis of the expression of IFN-α. *N* = 3, *p < 0.05, ^**^p < 0.01. Statistical comparisons were performed using two-way ANOVA with Tukey’s *post hoc* test **(B–D)** or unpaired Student’s *t*-test **(E,F)**.

To confirm whether the development of neuropathic pain caused by PTPRD was mediated *via* the STING-IFN-I pathway, we intrathecally administered animals with the STING-specific palmitoylation inhibitor H-151 at 7 days after CCI, when hypersensitivity had typically already improved in PTPRD knockdown mice (Figure 5A). As early as 4 h after the injection, the attenuation of hypersensitivity in shPTPRD animals was reversed and no differences in PWT, PWL, and PWF were observed between the control and shPTPRD + H-151 groups. This effect lasted for 48 h (Figures 5B–D). Therefore, H-151 administration disrupted the increased expression and secretion of IFN-α (Figures 5E,F), suggesting that PTPRD mediated neuropathic pain *via* the STING-IFN-I pathway in mice following CCI.

### Protein Tyrosine Phosphatase Receptor Type D Inhibitor 7-Butoxy Illudalic Acid Analog Alleviates Neuropathic Pain in Mice Following Chronic Constriction Injury

**FIGURE 6:**
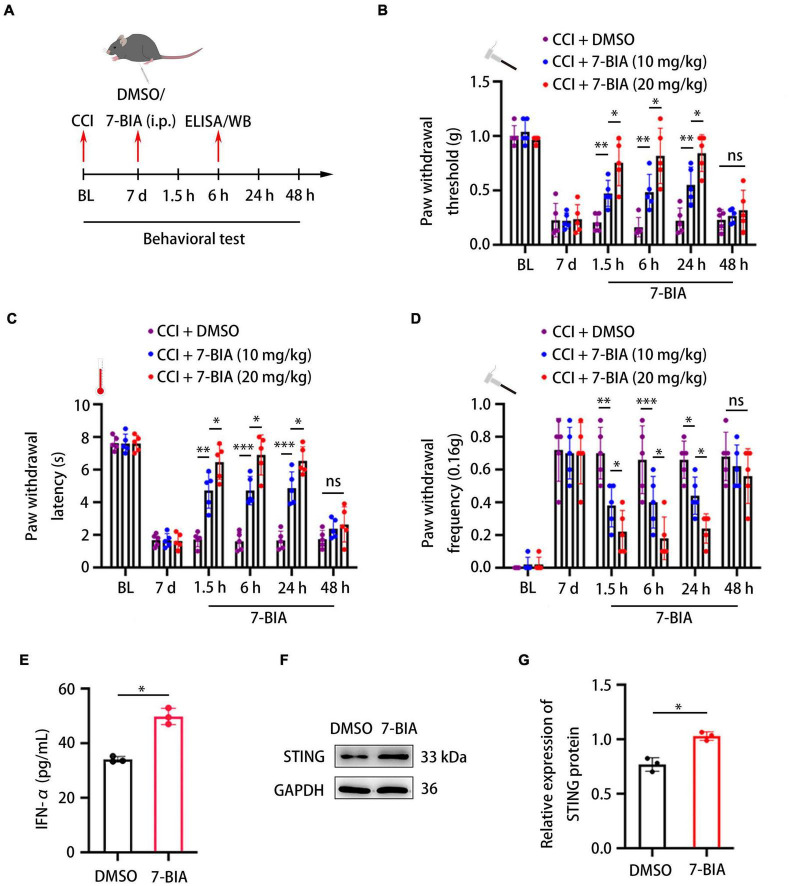
Protein tyrosine phosphatase receptor type D inhibitor 7-BIA alleviates neuropathic pain in mice following CCI. **(A)** Schedule of surgery, 7-BIA treatment (10 or 20 mg/kg, i.p.), behavioral testing, ELISA, and Western blotting. **(B–D)** Paw withdrawal threshold, paw withdrawal latency, and paw withdrawal frequency before CCI (BL), 7 days after the surgery, or 1.5, 6, 24, and 48 h after 7-BIA treatment. *N* = 5 mice per group, *p < 0.05, ^**^p < 0.01, ^***^p < 0.001. **(E)** ELISA analysis of the expression of IFN-α in the DRG after treatment with 7-BIA or DMSO. CCI + 7-BIA vs. CCI + DMSO, *N* = 3, *p < 0.05. **(F)** Western blot analysis of the expression of STING in DRGs treated with 7-BIA or DMSO. Experiment was repeated three times. **(G)** Quantitative analysis of data in **(F)**. CCI + 7-BIA vs. CCI + DMSO, *N* = 3, *p < 0.05. Statistical comparisons were performed using two-way ANOVA with Tukey’s *post hoc* test **(B–D)** or unpaired Student’s *t*-test **(E,G)**.

7-Butoxy illudalic acid analog is a small molecule drug recently synthesized to specifically target PTPRD as part of addiction therapy. Here, we investigated whether 7-BIA could also serve as a treatment for neuropathic pain. Seven days after CCI surgery, we intraperitoneally (i.p.) administered 7-BIA at 10 and 20 mg/kg and performed behavioral testing at 1.5, 6, 24, and 48 h after injection (Figure 6A). 7-BIA elicited a dose-dependent analgesic effect in CCI mice as early as 1.5 h after i.p. injection and lasted for 24 h, reducing both mechanical and thermal sensitivity (Figures 6B–D). Additionally, 7-BIA treatment resulted in increased DRG IFN-α levels after CCI (Figure 6E) as well as significantly increased protein levels of STING in the DRG (Figures 6F,G). Animals exhibited no changes in motor coordination (Supplementary Figure 2B). Together, these results suggested that PTPRD might be involved in the development of neuropathic pain, and its specific small molecule inhibitor 7-BIA could represent a novel and safe treatment for the clinical management of neuropathic pain without addiction risk.

## Discussion

Previous studies have reported an involvement of PTPRD in various central nervous system disorders, including addiction (Baron et al., 2010; Cohen and Mao, 2014), but there have been few reports on the role of PTPRD in the peripheral nervous system. A previous RNA-seq study found a significant upregulation of PTPRD 14 days after induction of a chronic pain model in rats, but it remained unknown whether this change in expression was biologically meaningful and whether PTPRD contributed to chronic neuropathic pain. In the present study, we found that PTPRD was typically located in the cytoplasm of Tuj1^+^ neurons in the DRG and confirmed a significant upregulation of PTPRD after CCI. Concomitantly with increased expression of PTPRD, we observed behavioral changes and found an increased sensitivity to mechanical and thermal stimuli, which suggested that PTPRD expression levels might be associated with the development of neuropathic pain following CCI. To confirm this hypothesis, we performed PTPRD knockdown using shRNA (shPTPRD) or the small molecule inhibitor 7-BIA, which revealed that a reduction of PTPRD levels following CCI effectively ameliorated neuropathic pain. Further investigating the mechanisms underlying the involvement of PTPRD in neuropathic pain, we found that PTPRD regulated this process *via* the STING-IFN-I pathway. Baseline PWT, PWL, and PWF levels were unaltered after PTPRD knockdown prior to CCI, but significantly increased (PWT, PWL) or reduced (PWF) after CCI surgery following shPTPRD treatment. DRG expression levels of PTPRD were low under physiological levels and therefore a knockdown may have had little influence on physiological function. Conversely, the analgesic effect was observed after CCI when expression of PTPRD was significantly upregulated and maintained at high levels. These results indicated that PTPRD might not participate in the regulation of pain sensitivity under physiological conditions, but instead was only involved in regulation of neuropathic pain following injury. There are many neuropathic pain models including surgical models like CCI, chemical pain models, and inflammatory pain models. Here, we investigated the role of PTPRD in the CCI-induced neuropathic pain, as CCI exhibits a similar pathophysiologic mechanism with carpal tunnel syndrome, which is the most common peripheral nerve chronic compression disease. However, it will be necessary to further test the function of PTPRD in other pain models in the later study.

Previous studies have shown that the development of neuropathic pain is closely related to neuroinflammation (Penas and Navarro, 2018; Yi et al., 2021). The expression of TNF-α, IL-6, and other pro-inflammatory factors in the DRG have been found to be increased after peripheral nerve injury (Sommer et al., 2018). Our results showed that IFN-α was significantly increased in DRG after both PTPRD knockdown or 7-BIA administration, while levels of IL-6, IL-1β, TNF-α, and IL-10 were not affected. IFN-α is a type I interferon which acts as an anti-inflammatory factor. Donnelly et al. (2021) previously found that intrathecal injection of IFN-α following CCI in mice prevented mechanical and thermal hypersensitivity. While these results indicated that PTPRD might regulate neuropathic pain *via* IFN-α. PTPRD is a phosphatase that is not thought to directly regulate the release of IFN-α. STING is a DNA receptor located in the intracellular endoplasmic reticulum (Rodero and Crow, 2016; Kwon and Bakhoum, 2020; Shi et al., 2021) and is involved in the regulation of IFN-α release in the DRG. Interestingly, the STING-IFN-I pathway has previously been shown to be a classic pathway involved in the regulation of neuropathic pain (Donnelly et al., 2021). We found that the expression of STING was significantly increased after either PTPRD knockdown or 7-BIA treatment, and therefore reasoned that PTPRD may influence IFN-α levels *via* STING. Further investigation of the relationship between PTPRD and STING will be of critical importance. PTPRD consists of an extracellular domain, a transmembrane protein structure, and an intracellular domain. The intracellular domain of PTPRD acts as a phosphatase which can lead to dephosphorylation of factors such as STAT3, PDGFRβ, and TBK1, amongst others (Tonks, 2006). Intriguingly, STING and TBK1 have been shown to form oligomers and STING can be phosphorylated by TBK1 (Bai and Liu, 2019). Based on these prior findings, we propose an indirect relationship between PTPRD and STING, for instance *via* TBK1, but this will need to be confirmed in future studies.

7-Butoxy illudalic acid analog is a small molecule drug targeting PTPRD that specifically inhibits the intracellular phosphatase activity of PTPRD (Uhl et al., 2018). Animal studies have confirmed the safety of 7-BIA, demonstrating repeated intraperitoneal injections were neither toxic nor addictive. Previous studies have shown that treatment of 7-BIA may relieve cocaine addiction in mice (Uhl et al., 2018). In our study, 7-BIA treatment significantly increased the PWT and PWL, while reducing the PWF following CCI in mice and exhibited an analgesic effect, consistent with results from PTPRD knockdown. The results of our study suggest that 7-BIA may represent a potential drug for the clinical management of neuropathic pain without addiction risk.

## Conclusion

In conclusion, we found a critical role for elevated DRG PTPRD levels in the development of CCI-induced neuropathic pain in mice. Our results suggest that PTPRD knockdown improved neuropathic pain *via* the STING-IFN-I pathway. A small molecule-specific inhibitor of PTPRD, 7-BIA, effectively prevented hypersensitivity after CCI and may represent a novel therapeutic agent for clinical management of neuropathic pain.

## Data Availability Statement

The original contributions presented in the study are included in the article/Supplementary Material, further inquiries can be directed to the corresponding authors.

## Ethics Statement

The animal study was reviewed and approved by the Institutional Animal Care and Use Committee of Jilin University.

## Author Contributions

CS and RC performed the main experiments, summarized the results, and wrote the manuscript. GW and ZZ analyzed the data. RC and SC provided the supervision and comments on the manuscript. All authors read and approved the final manuscript.

## Conflict of Interest

The authors declare that the research was conducted in the absence of any commercial or financial relationships that could be construed as a potential conflict of interest.

## Publisher’s Note

All claims expressed in this article are solely those of the authors and do not necessarily represent those of their affiliated organizations, or those of the publisher, the editors and the reviewers. Any product that may be evaluated in this article, or claim that may be made by its manufacturer, is not guaranteed or endorsed by the publisher.
