# Supplementary material for: Protein Tyrosine Phosphatase Receptor Type D Regulates Neuropathic Pain After Nerve Injury via the STING-IFN-I Pathway
Source: Front Mol Neurosci. 2022 Apr 14;15:859166. doi: 10.3389/fnmol.2022.859166 (PMC9047945; doi:10.3389/fnmol.2022.859166)
Supplement: Supplementary file 1 [file Data_Sheet_1.docx]

Supplementary Material


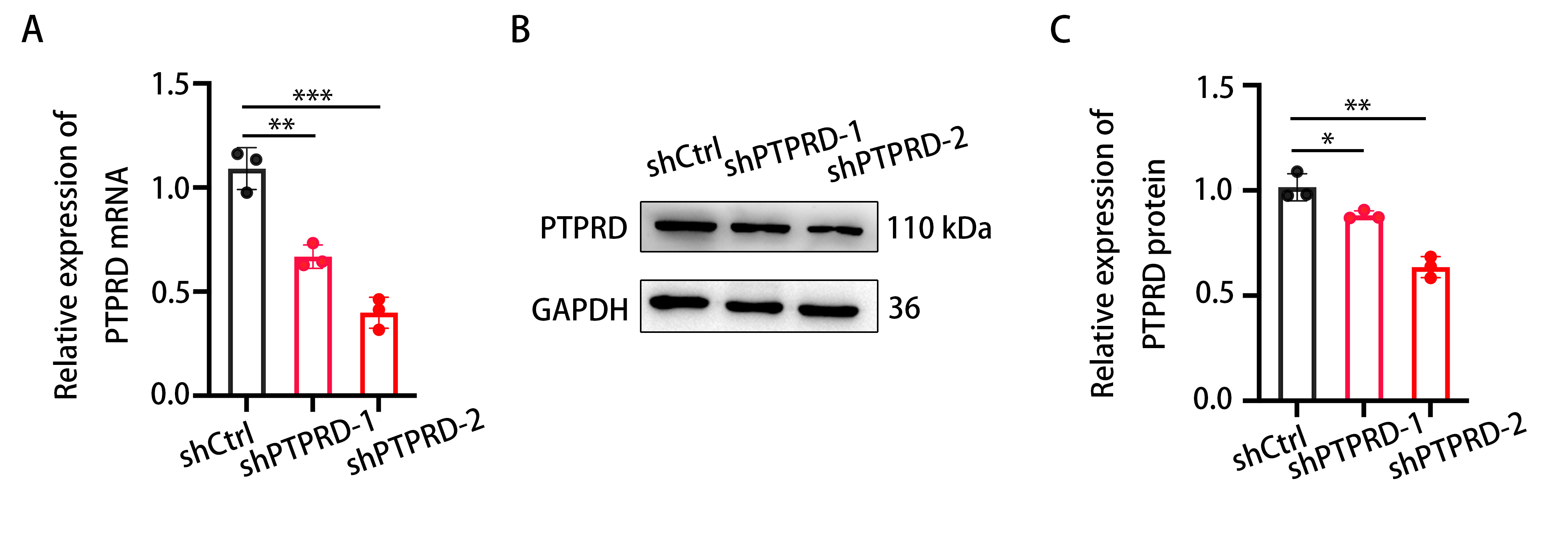


**Figure S1.** **Silence efficiency of two shPTPRDs.**

(A) qRT-PCR analysis of the relative expression of PTPRD in 293T cells transfected with shCtrl, shPTPRD-1 or shPTPRD-2. N = 3, ** p < 0.01, *** p < 0.001.

(B) Western blot of PTPRD in 293T cells transfected with shCtrl, shPTPRD-1 or shPTPRD-2. Each experiment was repeated three times.

(C) Quantitative analysis of data in (B). GAPDH served as the loading control. N = 3, * p < 0.05, ** p < 0.01.

Statistical comparisons were performed using unpaired student’s t test (A, C).


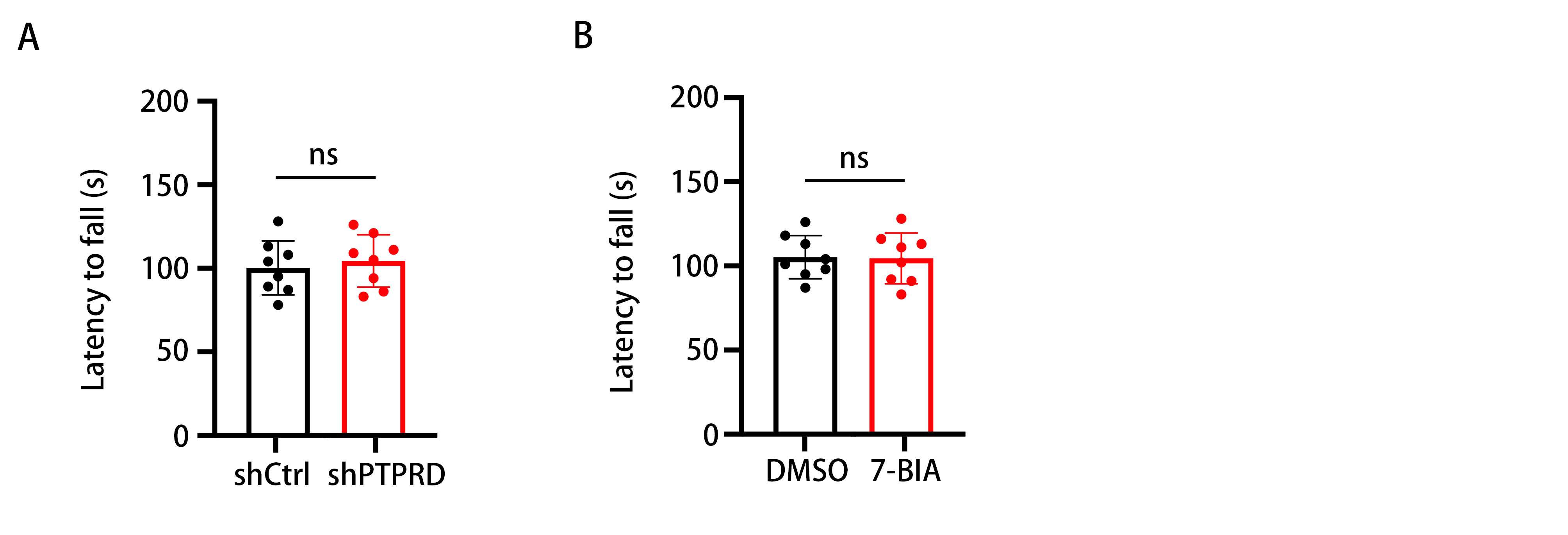


**Figure S2. Rotarod test of mice treated with shPTPRD or 7-BIA**

(A) The latency to fall in the rotarod test at 14 days after lentiviruses injection. N = 8 mice per group, p > 0.05.

(B) The latency to fall in therotarod test at 6 h after 7-BIA administration. N = 8 mice per group, p > 0.05.

Statistical comparisons were performed using unpaired student’s t test (A, B).

**Table.S1. Sequences of the primers for Quantitative real-time PCR**

| **Gene** | Sequences | Organism |
| --- | --- | --- |
| *PTPRD* | Forward:5’-GCCACGATCAGATACCATT-3’  Reverse: 5’-GCAGACAGACGGAAGTAG-3’ | Mus |
| *GAPDH* | Forward:5’-TGGAGTCTACTGGCGTCTT-3’  Reverse: 5’-TGTCATATTTCTCGTGGTTCA-3’ | Mus |
| *RIG-1* | Forward:5’-CTGCCTCACTCTTCCTCCAG-3’  Reverse: 5’-TGGCTTCACAAAGTCCACAG-3’ | Mus |
| *TRIF* | Forward: 5’-ATGGGCCCAGCAAGCTATGTAAC-3’  Reverse: 5’-TAGGGGAGGCTTGGAGGGATGGT-3’ | Mus |
| *STING* | Forward: 5’-AGATGTTTTCTGGGCAGACG-3’  Reverse: 5’-AATGACTTCAACCGGTTACTGG-3’ | Mus |
| *MAVs* | Forward: 5-’GAAAGCCCCAGTGCTGATCT-3’  Reverse: 5’-CACTTAGCCCAGGGCATTGA-3’ | Mus |
| *TLR-7* | Forward: 5’-TGCTGTGTGGTTTGTCTGGT-3’  Reverse: 5’-AAGTCACATCAGTGGCCAGG-3’ | Mus |
| *IL-6* | Forward: 5’-TGTGCAATGGCAATTCTGAT-3’  Reverse: 5’-GGTACTCCAGAAGACCAGAGGA-3’ | Mus |
| *IL-1β* | Forward: 5’-GCAACTGTTCCTGAACTCAACT-3’  Reverse: 5’-ATCTTTTGGGGTCCGTCAACT-3’ | Mus |
| *TNF-α* | Forward: 5’-AAGCCTGTAGCCCACGTCGT-3’  Reverse: 5’-AGGTACAACCCATCGGCTGG-3’ | Mus |
| *IL-10* | Forward: 5’-GGCAGAGAACCATGGCCCAGAA-3’  Reverse: 5’-AATCGATGACAGCGCCTCAGCC-3’ | Mus |
| *IFN-α* | Forward: 5’-CCTTCCTCCTGTCTGATGGA-3’  Reverse: 5’-ACTGGTTGCCATCAAACTCC-3’ | Mus |
